# Between defence and delivery: the DNA sensing response to gene electrotransfer

**DOI:** 10.2478/raon-2025-0063

**Published:** 2025-12-16

**Authors:** Tanja Jesenko, Masa Omerzel, Loree C Heller, Maja Cemazar

**Affiliations:** 1 Department of Experimental Oncology, Institute of Oncology Ljubljana, Ljubljana, Slovenia; 2 Faculty of Medicine, University of Ljubljana, Ljubljana, Slovenia; 3 Faculty of Health Sciences, University of Ljubljana, Ljubljana, Slovenia; 4 Department of Medical Engineering, University of South Florida, Tampa, Florida, USA; 5 Faculty of Health Sciences, University of Primorska, Izola, Slovenia

**Keywords:** gene electrotransfer, DNA sensors, gene therapy, immune response, plasmid DNA

## Abstract

**Background:**

Gene therapy has emerged as a transformative biomedical approach, offering new therapeutic possibilities from many so far uncurable diseases through the introduction of recombinant nucleic acids into target cells. Among non-viral delivery techniques, gene electrotransfer (GET) has become one of the frequently applied methods in clinical trials. It is based on the application of short, high-intensity electric pulses that transiently permeabilize cell membranes and enable the efficient transfer of plasmid DNA or other types of recombinant nucleic acids into various cell types. Beyond its role in gene delivery, GET can trigger complex cellular responses, as the introduced DNA interacts with intracellular DNA sensing pathways involved in innate immunity and inflammation. These responses can influence the therapeutic outcome – either by enhancing antitumour and vaccine-related immune activation or by reducing transfection efficiency when excessive inflammation or cell death occur. Our experimental findings in tumour, muscle, and skin models have shown that even non-coding plasmid DNA delivered by GET can induce local immune stimulation and tissue-specific inflammatory signaling, suggesting that the delivered DNA itself contributes to therapeutic efficacy.

**Conclusions:**

The dual nature of cellular responses following plasmid DNA GET represents both an opportunity and a challenge. Controlled activation of innate immunity can be harnessed to amplify antitumour or vaccine efficacy, while excessive responses may hinder applications requiring cell survival and sustained expression. Understanding these mechanisms enables the rational optimization of GET parameters and plasmid vector design to fully exploit the adjuvant effect or reduce the off-target effect of DNA sensing after GET, based on the desired application.

## Introduction

Gene therapy offers groundbreaking new opportunities for the treatment of various diseases. Several gene therapy medicines were approved in the last few years. Per the American Society for Gene and Cell Therapy (ASGTC) Q2 2025 Quarterly Data Report, 4,469 therapies are in development, ranging from preclinical through pre-registration, with oncology and rare diseases being the most targeted gene therapy areas. This report identifies 143 gene, cell and RNA therapies currently approved globally for clinical use.^1,2^ In oncology, gene therapy holds great promise in the treatment of cancer and can also be used for specific anti-tumour vaccination purposes.^3,4^

In general, a recombinant gene is a stretch of DNA that is created in the laboratory, bringing together DNA from different sources. Different methods can be used for the delivery of the recombinant genes. *In vivo* gene delivery methods are broadly divided into viral and non-viral categories. Each delivery type has applications in gene therapy, and each has associated problems. Viral delivery is effective; however, there are still some concerns, including the potential for insertional mutagenesis or induction of specific immune responses.5,6 However, due to the gained knowledge and evolvement of this technology, viral vectors are now generally considered as a safe delivery method.7,8 On the other hand, non-viral gene delivery is associated with low transfection efficiency, so chemical or physical assistance is often used, including lipid or polymer conjugation, particle-mediated delivery, hydrodynamic delivery, ultrasound or electroporation.^9–11^

Gene electrotransfer (GET), one of the most established non-viral methods for gene delivery, is based on the application of short electric pulses, which transiently permeabilize the cell membrane.^12^ This enables the efficient uptake of recombinant nucleic acids, including plasmid DNA, mRNA, and small interfering RNAs, into a wide range of cell types. GET has been widely applied in cancer gene therapy, partially for antiangiogenic therapy, but primarily for delivering plasmids encoding cytokines, such as interleukin-12 (IL-12), to stimulate strong antitumour immune responses, with several clinical trials demonstrating effectiveness of such approach.^13–15^ GET is also used in DNA vaccination, where it significantly enhances antigen expression and immune activation against infectious diseases. It also plays a role in the development of chimeric antigen receptor-T cell therapies (CAR-T), where electroporation is used *ex vivo* to insert genetic material, such as a construct or genome-editing tools into T cells as a non-viral alternative to viral vectors.^16^

**FIGURE 1.**
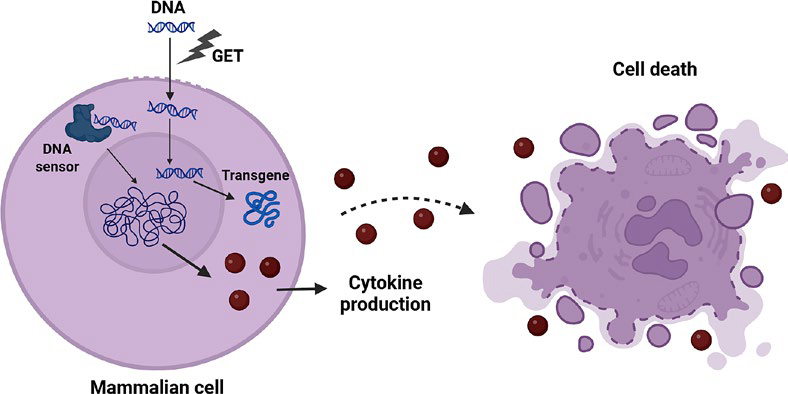
Delivery of DNA into the cells induces transgene expression as well as the activation of DNA sensing pathways, resulting in cytokine expression and cell death. Created in BioRender. Cemazar, M. (2025) https://BioRender.com/jazpf7t

Upon the delivery of the transgene product into the cells using viral or non-viral delivery methods, we can expect the on-target action of the delivered transgene as well as off-target cell-specific responses. These cellular responses evolved to maintain organismal homeostasis in response to the microbial infection. The innate immune system utilizes numerous germ-line encoded receptors termed pattern-recognition receptors (PRRs) to detect various pathogen-associated molecular patterns (PAMPs) and damage-associated molecular patterns (DAMPs).^17,18^ Nucleic acids can act as PAMP or DAMP, depending on its composition, origin or localization.^19,20^ When PRRs sense DNA, they are also called DNA sensors. The activation of these pathways results in innate antiviral immune responses in the form of proinflammatory cytokines and type I interferons and can also lead to cell death (Figure 1).^21,22^ On one hand, this activation can significantly impair the efficacy of gene therapy. On the other hand, the activation of the innate immune response can also be beneficial when the gene therapy is delivered to boost the anti-tumour immune response.^23^

In this review, we present the clinical applications of GET, outline the mechanisms underlying DNA delivery into cells, and discuss how the delivered DNA is recognized by cellular DNA-sensing pathways. Building on these insights, we further explore how such DNA sensing influences the current challenges and future perspectives of GET-based gene therapy.

### Clinical applications of GET

In the last decade, non-clinical research of GET has progressed into early human clinical trials, especially in the field of cancer vaccines and immunotherapy, but also for the vaccination against infectious disease. Numerous clinical trials, Phase 1–Phase 3, have been registered at ClinicalTrials database (ClinicalTrials.gov) and numerous reports already published in medical journals.

In cancer immunotherapy, intratumoral GET of plasmid encoding IL-12^14,24,25^ in patients with melanoma is most studied gene therapy (NCT01502293, NCT01502293, NCT05077033). IL-12 GET was safe, with no grade 3–4 toxicities and showed tumour necrosis, lymphocytic infiltration, and tumour regression even in non-injected lesions, implying a systemic immune stimulation and abscopal effect. IL-12 GET have also been combined with anti-PD-1 inhibitors (NCT03132675, NCT02493361) and studies suggested that adding intratumoral IL-12 GET may sensitize tumours to checkpoint inhibition by modifying the tumour microenvironment to become more immunogenic.^26,27^ The safety and effectiveness of combination treatment of IL-12 GET and pembrolizumab has also been proven in triple negative breast cancer (NCT03567720)^28^, however the treatment demonstrated ineffective in metastatic head and neck squamous cell carcinoma, leading to trial termination (NCT03823131). IL-12 GET demonstrated favourable safety profile and feasibility of therapeutic administration also in basal cell carcinoma.^13^

Anti-angiogenic therapy using GET for cancer treatment has been tested in clinical studies (NCT 01664273, NCT 01764009) with Antiangiogenic MEtargidin Peptide plasmid (AMEP)^15,29^, encoding an integrin-binding protein that inhibits tumour angiogenesis and proliferation. GET of plasmid AMEP was demonstrated feasible, safe (in a small cohort), and achieved local transfection of the plasmid. However, the trials were terminated due to insufficient patient enrolment following EMA approval of ipilimumab, as the inclusion criteria assumes exhaustion of all available treatment options.

Recent clinical studies explored a range of GET-delivered DNA-based cancer vaccines designed to induce targeted immune responses against tumour-associated antigens. For instance, the El-porCEA vaccine (NCT01064375) targets colorectal cancer using a plasmid DNA encoding carcinoembryonic antigen (CEA) fused with a tetanus toxoid helper epitope, while GX-188E (NCT01634503) and VGX-3100 (NCT03185013, NCT01304524) target HPV-16 and HPV-18 in cervical neoplasia.^30,31^ Similarly, INVAC-1 (NCT02301754)^32^ encodes the human telomerase reverse transcriptase (hTERT), over-expressed in most cancers, and INO-5150 (NCT02514213) combines prostate-specific antigen (PSA) and prostate-specific membrane antigen (PSMA) sequences to elicit prostate cancer immunity. Collectively, these trials demonstrate the versatility of DNA vaccines delivered via GET in oncology, showing favourable safety, tolerability, and immune activation across multiple tumour types.

GET is a promising method also for the delivery of DNA-based vaccines for infectious diseases. Several clinical studies have demonstrated the safety and tolerability of GET-delivered plasmids, encoding antigens, across infectious diseases, including HIV (PENNVAX™-B; NCT01082692, NCT02431767), influenza (VGX-3400X and H1/H5 formulations (NCT01142362, NCT03721978, NCT01405885), Ebola (INO-4201/4212, NCT02464670)^33^, and COVID-19 (INO-4800; NCT04447781, NCT04336410).^34^ GET promoted efficient antigen expression and robust immune responses, including both antibody and T-cell activation.

### Mechanisms of DNA delivery into cells via GET

The entry of DNA into mammalian cells during GET is a complex, multistep process involving the coordinated traversal of several biological barriers, the plasma membrane, cytoskeleton-rich cytoplasm, and nuclear envelope. The phenomenon underlying GET which induced the permeabilization of the first biological barrier, the plasma membrane, was first observed in the early 1970s, when short, high-intensity electric pulses were found to transiently increase the permeability of vesicular membranes.^35^ The extent and duration of this per-meabilization depend on the pulse amplitude, duration, and frequency, allowing controlled delivery of nucleic acids into the cells.^36,37^ The first successful demonstration of gene transfer by pulsed electric fields was reported by Neumann *et al*. in 1982, who introduced the herpes simplex thymidine kinase gene into mouse lyoma cells.^38^ Since then, GET has been widely used in both *in vitro* and *in vivo* applications for the transfer of various nucleic acids.^37^

**FIGURE 2.**
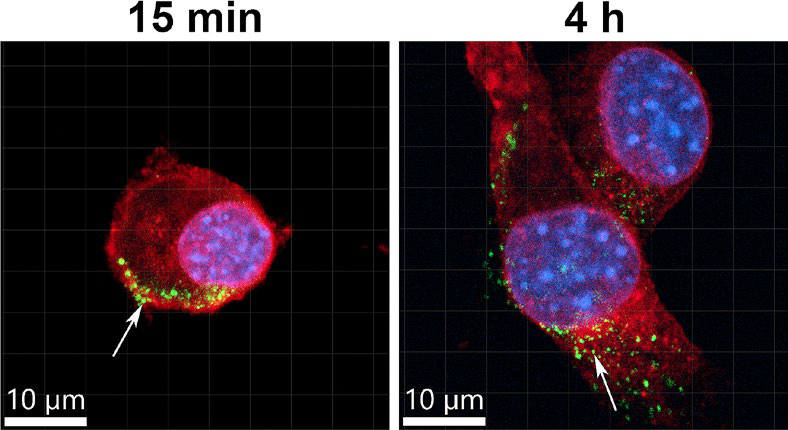
Localization of plasmid DNA after gene electrotransfer (GET) into C2C12 cell line, demonstrating DNA-membrane complexes after 15 min and internalization and presence in the cytoplasm and nucleus after 4 h. Plasma membrane is marked in red, nuclei in blue and plasmid DNA in green. Arrows represent plasmid DNA foci.

The GET process proceeds through several key stages: (1) plasma membrane electroporation, (2) DNA–membrane interaction, (3) DNA translocation across the membrane, (4) intracellular migration toward the nucleus, and (5) gene expression.^39^ Efficient transfection requires that each of these steps be successfully completed. Despite decades of research, the precise mechanisms of DNA uptake remain debated, particularly concerning the mode of transmembrane DNA transport. Two majors, not mutually exclusive, models have been proposed: the pore theory and the endocytosis theory. The pore theory posits that the applied electric field induces transient, nanometre-scale hydrophilic pores within the lipid bilayer, allowing charged macromolecules such as nucleic acids to pass into the cytosol.^40^ Although direct visualization of such pores has not yet been achieved, their existence is supported by mathematical modelling and the observed entry of otherwise impermeable fluorescent markers.^41,42^ Theoretical estimates suggest that pore diameters reach from 22.8 to 419 nm, and their lifetime is exceedingly short (on the order of 10 milliseconds) far shorter than the minutes-long timescale of plasmid DNA internalization.^41,43^ This discrepancy suggests that DNA uptake cannot occur solely through passive diffusion across transient pores. In contrast, the endocytosis theory is now supported by multiple experimental lines of evidence and proposes that DNA uptake occurs primarily via endocytic pathways. After application of electric pulses, plasmid DNA molecules are electrophoretically driven toward the plasma membrane, where they form stable DNA-membrane complexes that subsequently undergo internalization (Figure 2).^42,43^ Pharmacological inhibition and RNA interference studies have demonstrated that clathrin-mediated and caveolin/raft-mediated endocytosis are major contributors to plasmid DNA uptake, together accounting for approximately 75% of internalized DNA.^44^ This theory was also demonstrated in vivo in the mouse muscle tissue using an endocytosis inhibitor, showing that endocytosis is the main mechanism of entrance of DNA after GET, which leads to the production of the transgene.^45^

Following internalization, DNA must traverse the cytoplasm to reach the nucleus (Figure 2) a journey hindered by the dense cytoskeletal network. DNA aggregates were demonstrated to be actively transported by the actin and the microtubule networks.^46,47^ An *in vitro* study has indicated that DNA delivered via GET uses the classical endosomal trafficking pathways as plasmid DNA trajectories were co-localized with the biomarkers for endosomes.^48^

The last and the most challenging barrier is the nuclear membrane. The exact mechanism by which plasmid DNA crosses the nuclear barrier remains incompletely understood. The plasmid DNA must first escape the endosomes to be release into the cytosol to be able to enter the nucleus and be transcribed. Typically, macromolecules traverse the nuclear envelope through nuclear pore complexes (NPCs), which allow passive diffusion of small molecules (<9 nm or ~40 kDa) and active transport of larger molecules up to 39 nm when equipped with nuclear localization signals (NLSs).^49^ However, plasmid DNA, with a radius of gyration of approximately 100 nm, is far too large for passive diffusion through NPCs and the presence of NLSs is not ensured in all DNA constructs. Therefore, the most straightforward mechanism is reached in dividing cells. During mitosis, the nuclear envelope breaks down and facilitates passive nuclear entry of plasmid DNA during the formation of nuclear envelope in anaphase of mitosis, which can be enhanced by synchronizing cells at the G2–M phase before electroporation or transfection.^50^

Taken together, these findings indicate that at every stage of the GET process, plasmid DNA can transiently reside within the cytoplasmic compartment, where it may be detected by cellular DNA sensors. Although the majority of DNA internalization (~75%) proceeds via endocytic and endosomal pathways that deliver the cargo close to the perinuclear region, a significant proportion – approximately 25% – likely enters the cytosol through alternative routes.^47^ Furthermore, even DNA internalized by endocytosis can become cytosol-accessible following endosomal escape, a critical step for efficient transgene expression. This transient cytosolic presence of exogenous DNA provides the molecular basis for its recognition by intracellular DNA-sensing pathways.

### Intracellular DNA sensors

The immune-stimulatory role of nucleic acids is well established. Its immunogenicity was first described in immune cells. The most well-characterized DNA sensor is Toll-like receptor 9 (TLR9), which is found predominantly in the endosomes of immune cells and detects mainly CpG motif DNA and RNA-DNA hybrids.^51,52^ In contrast, other intracellular DNA sensors are nuclear or cytosolic, are ubiquitous and present in virtually any mammalian cell, including tumour cells, and detect various DNA motifs.^17,53^

The DNA sensors are germline-encoded and function in the detection of intracellular pathogens, including viruses. Although viral proteins may trigger specific PRRs, the predominant viral components activating them are nucleic acids.^54^ Viruses, including viral vectors commonly used for gene therapy applications, trigger these pathways. For example, adenoviruses activate multiple pattern recognition pathways^55–57^, while adeno-associated viruses show significant, but reduced production type I interferon mRNA when compared to adenovirus^58^, although the innate immune responses can influence the outcome of these gene therapies.^59^ HIV1, upon which lentiviral gene therapy vectors are based, inhibit the host’s type I interferon response at several levels.^60^ These host-directed viral activities reduce, but do not completely reverse the production of inflammatory molecules.

There are multiple DNA sensing pathways identified, and the list of newly recognized DNA sensors grows every year.^22,53,61,62^ Their ligands and signalling cascades are incompletely characterized; however, cytosolic DNA sensor binding is known to control the production of pro-inflammatory cytokines and interferons.^17,62^ Their activation can also lead to the induction of cell death that elicits inflammation.^22^ DNA-dependent activator of interferon regulator factor (ZBP1), was the first intracellular DNA sensor described.^63^ The observation that ZBP1 deficient mice responded similarly to DNA vaccination as wild type mice suggested that redundant DNA sensors might exist. Subsequently, several groups reported that DNA binding to absent in melanoma 2 (AIM2) induces caspase-1 activation, leading to the secretion of interleukin-1 beta (IL-1β) and interleukin 18 (IL-18).^64,65^ DNA binding and activation of the AIM2-like protein gamma-interferon-inducible protein Ifi-16 (IFI16; mouse orthologue ifi204) mediates interferon beta (IFNβ) production, particularly in myeloid cells.^66^ Several members of the DexD/H-box helicases (DDX) family bind DNA ligands and induce pro-inflammatory cytokine production. DDX60 binds both RNA and DNA^67^ and is required for RIG-1/DDX58 upregulation of Type I interferon gene expression.^68^ RIG-I can be activated indirectly by cytosolic DNA when RNA polymerase III transcribes DNA into its activator, dsRNA containing a 5’ triphosphate.^69^ Additional DNA binding proteins putatively mediating inflammatory have been described. Binding of DNA to Ku70, a component of DNA protein kinase induces the production of Type III, not Type I interferon.^70^ IFNβ is produced after DNA binding by cyclic GMP-AMP synthase (cGAS).^71^ The synthesized cGAMP acts as a second messenger required for STING (stimulator of IFN genes)-dependent IFNβ production. Recent literature highlights STING as the central regulator of cytosolic DNA sensing and downstream innate immune signalling, orchestrating the induction of type I interferons and proinflammatory cytokines.^72,73^ The readers can find more extensive knowledge on DNA sensors in other reviews.^20,22,62^

### Activation of DNA sensing after plasmid DNA GET

Non-viral gene therapies induce cells to produce inflammatory molecules, and this effect was extensively studied by our research group after plasmid DNA GET.^74–79^ In our experiments, we found that after GET of non-coding plasmid DNA, some mouse melanoma ^75,77,80^ and sarcoma tumours^81^ completely regressed. Similar observations were also made by other groups investigating *in vivo* GET.^82–87^ Tumour regression required the presence of both electric pulses and DNA, although inclusion of a therapeutic gene was not essential. The extent of regression correlated with the plasmid DNA concentration and the specific electric pulse parameters applied. Elevated levels of inflammatory proteins were associated with this regression, indicating that inflammation may play a contributory role.^88^

Because GET introduces DNA into both the cytosol and endosomes of the cell, we wondered whether the observed inflammation results from activation of the well-established endosomal PRR TLR9 or from the more recently identified cytosolic DNA sensors in tumour cells. Because DNA sensors are expressed across various cell types, the profile of downstream regulated proteins likely depends on the cellular composition of the target tissue. We have demonstrated that, while TLR9 mRNA levels were unchanged after DNA GET, mRNA levels of specific DNA sensors (ZBP1, ddx60 and ifi204) and IFNβ1 were upregulated in tumour cells, which could be the mechanism behind the observed anti-tumour effects of DNA GET.^76–78^ We have demonstrated this observation in WEHI164 fibrosarcoma cells, TS/A mammary adenocarcinoma cells^76^ and in B16F10 melanoma cells^78^, spheroids^89^ and tumours.^75,77,80,88^ B16F10 tumours respond to plasmid DNA GET with the production of several pro-inflammatory cytokines and chemokines and ifi204 mRNA upregulation.^77^

When comparing GET with an empty plasmid to that with a plasmid encoding IL-12 in B16F10 and CT26 tumour cells, we observed that both plasmids induced an increase in mRNA levels of several DNA sensors, many of which are associated with cell death, most prominent among them DDX60 and ZBP1.^79^ However, cytokine profiling showed that some cytokines were expressed only after GET with the therapeutic IL-12 plasmid.^79^ This indicates that the cellular response to plasmid GET is multilayered, involving activation of multiple signalling cascades. Importantly, IL-12 GET led to the induction of two inducible damage-associated molecular patterns (iDAMPs), IL-6 and TNF-α. The presence of these iDAMPs suggests that therapeutic GET provides a dual advantage: not only are constitutive DAMPs released from dying cells, but tumour-resident antigen-presenting cells such as dendritic cells and macrophages can also be activated by iDAMPs, thereby promoting efficient CD8^+^ T-cell cross-priming within the tumour microenvironment.^90,91^

Further, we were also interested in the applications of DNA GET in muscle or in the skin for applications of DNA vaccination. C2C12 myoblasts reacted robustly to backbone plasmid DNA GET and the effect was more pronounced as in tumour cells. IFNβ mRNA was upregulated four hours after GET and protein levels mirror this upregulation, suggesting DNA sensor activation.^74^ This correlated with significant increases in the mRNAs of DNA sensors ZBP1, DDX60 and ifi204. Other DNA sensors, specifically DDX41, DHX9, DHX36, Ku70, MRE11, PQBP1 and cGAS, were detected in these cells but not upregulated, while RIG-I and TLR9 were not detected. This mRNA upregulation was also reflected in protein levels. An increase in IFNβ mRNA also occurred after delivery using another method of non-viral delivery, a non-liposomal formulation comprised of a lipid and a protein/polyamine mixture. This upregulation was paralleled by the upregulation of a similar repertoire of DNA sensors mRNAs, supporting the concept that this effect is not limited to GET and is universal after gene delivery.^74^ In myoblasts we also demonstrated that upregulation of mRNAs and proteins do not necessarily predict DNA detection and binding. We revealed early events upon plasmid DNA entrance into the cell and identified ZBP1, ifi204, and DHX9 as early plasmid DNA binding proteins.^74^

Since myoblasts in culture respond strongly to plasmid DNA GET with the DNA sensor-dependent production of pro-inflammatory proteins, it is possible that this pathway is responsible for the inflammation observed after intramuscular delivery *in vivo*. Inflammation associated with DNA GET was demonstrated to increase vaccine therapeutic efficacy and this effect was initially described nearly 20 years ago.^92–94^ The activation of the innate immune response can therefore influence the vaccination efficiency and should be considered when developing gene therapy drug products for vaccination purposes. This response is not limited to DNA delivery by GET but is rather ubiquitous to all types of viral and non-viral delivery methods that deliver nucleic acids into the cell cytosol.

Building on these findings, we extended our investigation of DNA sensing to the skin as an organ for DNA vaccination. The skin is the body’s largest and one of its most immunologically active organs, serving not only as a physical barrier but also as an interface rich in innate and adaptive immune elements. Its complex cellular architecture comprising keratinocytes, fibroblasts, and immune cells makes it a particularly suitable site for GET, where the delivery of plasmid DNA can trigger diverse intracellular signalling pathways depending on cell type. Our studies demonstrated that noncoding plasmid DNA GET activates cytosolic DNA sensing mechanisms in skin cells, similar to those previously observed in muscle and tumour models. Specifically, qPCR analysis revealed the upregulation of DDX60, AIM2, ZBP1, ifi202, and ifi204 mRNAs in keratinocytes, and of DDX60, ZBP1, and ifi204 in fibroblasts.^95^ These transcriptional changes were accompanied by increased production of cytokines and chemokines, confirming a strong innate immune activation following DNA GET. In vivo experiments in mouse skin further supported these results, showing elevated expression of DNA sensor mRNA and pro-inflammatory cytokines IFN-β1, TNFα and Il-1β. IFN-β1 and TNFα were immunohistologically detected in fibroblasts, keratinocytes and macrophages in skin which correlated with our observed gene and protein expression *in vitro* and *in vivo*. In contrast, Il-1β was detected in keratinocytes and macrophages but not in fibroblasts, which also correlated with *in vitro* results. Thus, immunofluorescent staining identified keratinocytes, fibroblasts, and macrophages as principal contributors to the local immune response to plasmid DNA GET.

### The ambivalent role of DNA sensing in plasmid DNA GET

**FIGURE 3.**
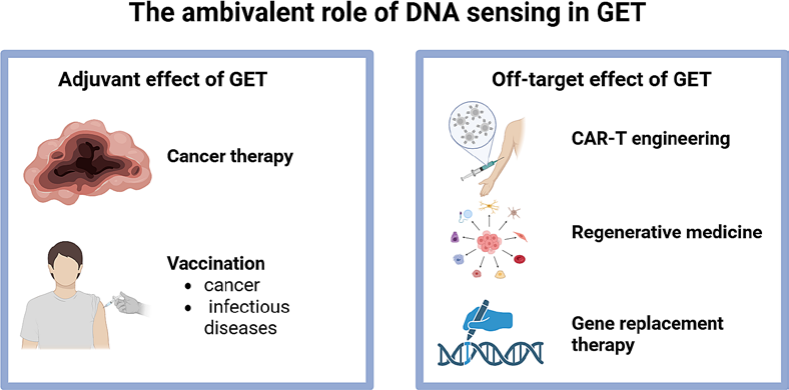
Ambivalent role of DNA sensing in plasmid DNA gene electrotransfer (GET), acting as a positive or negative ally. Created in BioRender. Cemazar, M. (2025) https://BioRender.com/zqbdsxi

The widespread presence of DNA sensors across mammalian cell types underscores the complex and context-dependent nature of the innate immune response to plasmid DNA GET. Tumour, muscle, and skin cells each exhibit distinct transcriptional and cytokine response profiles following DNA delivery, reflecting tissue-specific engagement of cytosolic DNA sensing pathways. This cellular diversity presents both an opportunity and a challenge on one hand, it allows for the exploitation of tissue-specific immunogenicity to enhance therapeutic outcomes, while on the other, it complicates the prediction of the *in vivo* responses. Collectively, current evidence indicates that the antitumour and immunostimulatory effects of plasmid DNA GET are not entirely dependent on the encoded therapeutic gene but can arise intrinsically from the introduction of nucleic acid itself. This intrinsic immunogenicity, mediated through activation of DNA sensors and subsequent cytokine production, can be regarded as an adjuvant effect that potentiates immune activation, leading to more potent antitumour or vaccination effects (Figure 3).

However, this activation not only triggers a strong innate immune response but also leads to various forms of cell death, depending on the cell type and intensity of stimulation. While cell death is highly advantageous in the context of antitumour therapy, where the release DAMPs and cytokines enhance immune activation and tumour clearance, it poses a significant limitation in applications where cell survival and functionality are required, such as engineering of CAR-T cells, regenerative medicine or gene replacement therapy (Figure 3).96-98 A notable example is CAR-T cell therapy, in which efficient gene delivery must be achieved without compromising T-cell viability, proliferation, and effector function. In this setting, excessive activation of DNA sensing pathways or strong GET parameters can impair cell viability and reduce therapeutic potency. Therefore, careful optimization of both the vector design to minimize immunostimulatory motifs and improve expression efficiency and the electric parameters to balance membrane permeability and cell survival is essential to ensure successful transfection while preserving cell viability.

## Conclusions

In summary, DNA sensing pathway activation represents both an opportunity and a challenge in GET-based applications. When harnessed appropriately, the resulting inflammation and cell death can act as an intrinsic adjuvant effect, amplifying the therapeutic outcome in cancer treatment and DNA vaccination. Conversely, when gene delivery aims to produce functional proteins in viable cells, as in CAR-T cell engineering, gene replacement therapy or regenerative medicine, such immune activation and cytotoxicity may become detrimental. It is therefore critical to tailor gene delivery strategies to the intended biological goal leveraging the adjuvant properties of DNA sensing in immunogenic applications, while minimizing off-target effects and preserving cell viability in cases requiring sustained cellular function.
